# *TOLLIP* gene variant is associated with *Plasmodium vivax* malaria in the Brazilian Amazon

**DOI:** 10.1186/s12936-017-1754-7

**Published:** 2017-03-13

**Authors:** Larissa W. Brasil, Laila R. A. Barbosa, Felipe J. de Araujo, Allyson G. da Costa, Luan D. O. da Silva, Suzana K. Pinheiro, Anne C. G. de Almeida, Andrea Kuhn, Sheila Vitor-Silva, Gisely C. de Melo, Wuelton M. Monteiro, Marcus V. G. de Lacerda, Rajendranath Ramasawmy

**Affiliations:** 1 Diretoria de Ensino e Pesquisa, Fundação de Medicina Tropical Dr. Heitor Vieira Dourado, Av. Pedro Teixeira, 25-Dom Pedro, Manaus, Amazonas CEP:69040-000 Brazil; 2 Escola Superior de Ciências da Saúde, Universidade do Estado do Amazonas (UEA), Manaus, AM Brazil; 3 Instituto de Pesquisas Leônidas & Maria Deane, FIOCRUZ, Manaus, AM Brazil; 4 Faculdade de Medicina, Universidade Nilton Lins, Manaus, AM Brazil; 5 Programa de Pós-Graduação em Imunologia Básica e Aplicada Universidade Federal do Amazonas (UFAM), Manaus, AM Brazil

**Keywords:** Malaria, *Plasmodium vivax*, *TOLLIP* gene, Amazon

## Abstract

**Background:**

Toll-interacting protein is a negative regulator in the TLR signaling cascade, particularly by impeding the TLR2 and, TLR4 pathway. Recently, TOLLIP was shown to regulate human TLR signaling pathways. Two common *TOLLIP* polymorphisms (rs5743899 and rs3750920) were reported to be influencing IL-6, TNF and IL-10 expression. In this study, *TOLLIP* variants were investigated to their relation to *Plasmodium vivax* malaria in the Brazilian Amazon.

**Methods:**

This cohort study was performed in the municipalities of Careiro and, Manaus, in Western Brazilian Amazon. A total of 319 patients with *P. vivax* malaria and, 263 healthy controls with no previous history of malaria were included in the study. Genomic DNA was extracted from blood collected on filter paper, using the QIAamp^®^ DNA Mini Kit, according to the manufacturer’s suggested protocol. The rs5743899 and rs3750920 polymorphisms of the *TOLLIP* gene were typed by PCR–RFLP.

**Results:**

Homozygous individuals for the rs3750920 T allele gene had twice the risk of developing malaria when compared to individuals homozygous for the C allele (OR 2.0 [95% CI 1.23–3.07]; p = 0.004). In the dominant model, carriers the C allele indicates protection to malaria, carriers of the C allele were compared to individuals with the T allele, and the difference is highly significant (OR 0.52 [95% CI 0.37–0.76]; p = 0.0006). The linkage disequilibrium between the two polymorphisms was weak (r^2^ = 0.037; D′ = 0.27).

**Conclusions:**

These findings suggest that genes involved in the TLRs-pathway may be involved in malaria susceptibility. The association of the *TOLLIP* rs3750920 T allele with susceptibility to malaria further provides evidence that genetic variations in immune response genes may predispose individuals to malaria.

## Background

Malaria, a health burden in the Amazon region of Brazil, is responsible for 99.9% of all reported malarial cases in Brazil and nearly 140,000 cases were registered the region in 2015 [1]. The socioeconomic and, environmental conditions in the Amazon favor the proliferation of the vector, the mosquito *Anopheles darlingi*. In the State of Amazonas, 75,575 cases were reported in 2015. *Plasmodium vivax* accounts for 90% and, *Plasmodium falciparum* is responsible for the majority of the remaining cases [2]. *Plasmodium vivax,* in contrast to *P. falciparum,* has a dormant form in the liver, the hypnozoite, which subsequently causes new infections in the blood known as relapses, and this represents a real challenge for vivax malaria eradication [3].

Several studies have linked high levels of TNF, IL-1β, IL-2, IL-4, IL-6, IL-8, IL-10 and IL-12 cytokines with *P. vivax* infections [4–7]. Severe malaria patients display increase levels of pro-inflammatory cytokines, TNF, IL-6 and IFN-γ [8–11] while acute malaria individuals show high levels of the anti-inflammatory cytokines IL10 [12–14].

Innate immunity is defined as the first line of host defense to invading pathogens. Toll-like receptors recognize invading pathogens and, TLR-2 and, TLR-4 sense the glycosylphosphatidylinositol (GPI) of *Plasmodium* [15]. Mice immunized with synthesized GPI and challenged with *Plasmodium berghei* are protected against malaria-related acidosis and, pulmonary oedema, suggesting that GPI may contribute to malaria mortality and, pathogenesis [16]. In vitro, GPI influences the expression of adhesion molecules and, the pro-inflammatory cytokines IL-1 and TNF [17].

The above studies show the importance of the TLR pathway in *Plasmodium*-infection. Overexpression of Toll-interacting protein (TOLLIP) leads to the impairment of the activation of the transcription factor NF-κB and, thus limits the production of pro-inflammatory mediators [18]. TOLLIP is also suggested to inhibit TLR-mediated cellular responses by associating directly with TLR2 and, TLR4 to suppress the phosphorylation and kinase activity of IL-1 receptor associated Kinase [19]. TOLLIP skews the pro-inflammatory cytokines response of TLR-2 and, TLR-4 into an increased IL-10 and, decreased IL-6 expression in human peripheral blood monocytes [20]. IL-6 and TNF are key mediators associated with malarial symptoms and their levels are proportional to the severity of the disease [21]. Several variants of the *TOLLIP* gene have been shown to be associated with tuberculosis and its transcription levels [20], cutaneous leishmaniasis [22] and, leprosy [23].

The role played by the TLR pathway in keeping in check parasite multiplication in animal models and the suggestion that *TOLLIP g*ene may downregulate TLR-2 and, TLR-4 lead us to hypothesize that variants of the *TOLLIP* gene may be associated with malaria caused by *P. vivax* in the state of Amazonas, Brazil. To this end, a case–control study was conducted by investigating two common variants rs5743899 and rs3750920 that were previously reported to probably influence the expression of TNF, IL-6 and, IL-10 in patients with malaria and healthy controls from the same endemic areas followed for a period of 12 months. The authors report that the *TOLLIP* rs3750920C allele is associated with protection to malaria while the T allele with susceptibility.

## Methods

### Site of study and patients

The present study was conducted from a cohort studies in the municipality of Careiro (in the central region of the Amazon State) and, in the peri-urban region of Manaus (Brasileirinho, Ipiranga and Puraquequara communities), the capital city of the Amazonas state, Brazil. These regions have been invaded over the years by destroying the forest for settlements, agriculture and, farming.

Manaus has a population of approximately 2,020,301 inhabitants. Most of them live in urban/peri-urban area where there has been an intense migration process, combined with precarious epidemiological and, entomological surveillance resulting in the active transmission of malaria. The IPA in the city ranges from low to medium risk in rural areas and, between the peri-urban areas varies from no risk to high risk.

The municipality has an average of 75% of malaria caused by *P. vivax* reported in the last 5 years, which is very similar to the percentage of infections by *P. vivax* reported in other areas of the Brazilian Amazon. In the last 5 years there has been some seasonality to malaria despite the stable transmission, with most reported cases during the period from May to September.

The communities of Brasileirinho, Ipiranga and, some extension Puraquequara, are located in the peri-urban area, east of the city of Manaus. The city’s boundary is surrounded with forest and has suffered deforestation and, gradually becomes endemic areas of malaria. In the city of Manaus, these areas have become vulnerable to malaria transmission, despite the decline in IPA observed in recent years. The subsistence activities of the population are concentrated in the agricultural sector and, extraction. Many of the residents of these areas, however, work in the city of Manaus and, thus travel daily to Manaus. It is noteworthy that in this location there are several farms used for recreation and, religious retreat, where many residents of Manaus go to spend their weekends and, holidays. These fluxes can increase the risk of malaria transmission. The occurrence of malaria in this population is common. Basic sanitary is lacking in these communities. There is no garbage collection. Drinking water is from wells or streams.

The Municipality of Careiro currently has an estimated population of 30,000 inhabitants, most of them living in rural areas, supported by federal programs that encourage the practice of agriculture. This population has been previously described elsewhere [24].

These areas have become endemic particularly for *P. vivax*. These populations were chosen for study based on low migration of its inhabitants (intra-regional) and, similar profile of rural malaria transmission. All of the case patients included in the study are patients with symptomatic malaria, diagnosed in the health post of municipalities and, are provided with malarial treatments following detection of the parasites.

A total of 319 patients with malaria confirmed by direct microscope examination of Giemsa-stained specimens for the presence of *P. vivax* parasites and, 263 healthy controls with no history of malaria are included in the study. Basic characteristics of the study population are shown in Table 2.

### Molecular characterization

Genomic DNA was extracted from blood collected on filter paper, using the QIAamp^®^ DNA Mini Kit (QIAGEN^®^, Germany), according to the manufacturer’s suggested protocol.

**Fig. 1:**
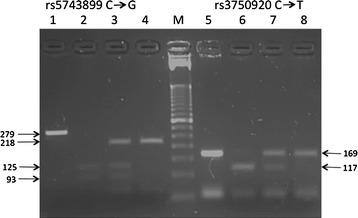
The rs5743899 and, rs3750920 polymorphisms of the *TOLLIP* gene as detected by restriction enzymes *Hha*I and, *Msp*I respectively. The gel depicts the PCR product (*lane 1*), restriction patterns related to homozygozity GG (*lane 2*), heterozygozity GA (*lane 3*) and, homozygosity AA (*lane 4*) for the rs5743899 and, similarly for the rs3750920, PCR product (*lane 5*), homozygote CC (*lane 6*), heterozygote CT (*lane 7*) and, homozygote TT (*lane 6*). *Lane M*, DNA molecular-weight marker ladder 50 base pairs

Genotypic determinations of the rs5743899 and rs3750920 located in the intron and exon 4 respectively of the *TOLLIP* gene were performed by PCR–RFLP as described elsewhere [22]. Briefly, the following pairs of primers: rs5743899F: 5′-GGC AAT GGC AGT GGC CAC CAG TGA-3′ and rs5743899R: 5′-CCG ATGCCC GCA CAC CTG TGT GAT-3′ for (rs5743899) and rs3750920F: 5′-AGG CGT GCA GCTCAC CGC GTA GGA-3′ and rs3750920R: 5′-GAG AGC CTT CTC CAT GGA CGA CCG C-3′ for (rs3750920) flanking the polymorphisms, were used to amplify separately a stretch of DNA of 279 and 169 bp respectively and, digested by the corresponding restriction enzymes *Hha*I and, *Msp*I, with the fragment-size separated by electrophoresis in 3% agarose gel (Fig. 1).

### Data analysis

Statistical analysis was performed using the website http://ihg.gsf.de/cgibin/hw/hwa1.pl for logistic regression analysis. Two-tailed χ^2^ test along with the odds ratio (OR) and, 95% confidence interval [CI] was applied for comparison of patients with malaria to healthy controls. Allelic and genotypic frequencies were calculated by gene counting directly. Hardy–Weinberg equilibrium (HWE) was calculated by χ^2^ test by comparing the observed number of different genotypes frequency with those expected under HW expectation. The software Haploview 4.2 was used for calculating Linkage disequilibrium between the rs5743899 and, rs3750920.

## Results

Previous study of surveillance in the Careiro, Brasileirinho, Ipiranga and, Puraquequara communities showed that a significant proportion of the population reported previous infection with *P. vivax* and/or *P. falciparum*, indicating that most individuals were exposed to the malaria parasite along the years. The baseline characteristics of the individuals participating in this study are shown in Table 1. A total of 319 had malaria and, 263 healthy controls were recruited from the same endemic area. Approximately 60% were male with a mean age of 38 years [Careiro: 35.9 (SD 17.8); Brasileirinho + Ipiranga + Puraquequara: 39.5 (SD 21.3)]. Among individuals with malaria, male has a higher frequency of malaria. Most of them reported previous history of the disease (Careiro: 65%; Brasileirinho + Ipiranga + Puraquequara: 84%), with an average of five episodes in Careiro and, ten episodes in Brasileirinho + Ipiranga + Puraquequara.

**Table 1:** Clinical and demographic characteristics of patients with and without *P. vivax* malaria

Clinical and demographic characteristics	*P. vivax* malaria patients	Controls
	n = 111	N = 97
Careiro		
Gender		
Male n (%)	63 (57)	61 (63)
Female n (%)	48 (43)	36 (37)
Age/years (mean ± SD)	35.9 ± 17.8	34.2 ± 16
Previous history of malaria n (%)	72 (65)	–
Episodes of malaria (mean)	05	–
*Plasmodium vivax* (%)	81	–
*Plasmodium falciparum* (%)	19	–

Clinical and demographic characteristics	*P. vivax* malaria patients	Controls
	n = 208	n = 166
Brasileirinho + Ipiranga + Puraquequara		
Gender		
Male n (%)	131 (63)	84 (51)
Female n (%)	77 (37)	82 (49)
Age/years (mean ± SD)	39.5 ± 21.3	39.5 ± 21.3
Previous history of malaria n (%)	174 (84)	–
Episodes of malaria (mean)	10	–
*Plasmodium vivax* (%)	89	–
*Plasmodium falciparum* (%)	11	–

These individuals were genotyped for the rs5743899 and, rs3750920 of *TOLLIP* gene. The distribution of genotypes and, allele frequencies of both polymorphisms are shown in Table 2. Both polymorphisms were in Hardy–Weinberg equilibrium among the healthy controls while the rs3750920 deviate slightly among patients with malaria (p = 0.02). No difference was observed for the rs5743899 genotypes and, alleles distributions between patients with malaria and, healthy controls. In contrast, the genotypes distributions of the rs3750920 differed significantly (p = 0.0024). Among the patients with malaria, homozygosity for the T allele was prevalent 37 vs. 24% and, had twice the risk of developing malaria when compared to individuals homozygous for the C allele (p = 0.004; OR 2.0 [95% CI 1.23–3.07]). The C allele indicates protection to malaria. In the dominant model, carriers of the C allele were compared to individuals with the T allele and, the difference is highly significant (p = 0.0006; OR 0.52 [95% CI0.37–0.76]). Comparison of heterozygous individuals (CT) to homozygous individuals for the T allele also showed a similar trend (p = 0.002; OR: 0.54 [95% CI 0.36–0.80]). The linkage disequilibrium between the two polymorphisms is weak as calculated by the Haploview 4.2 program. The r^2^ and, D′ are 0.037 and, 0.27 respectively.

**Table 2:** Genotype and allele frequencies for the single nucleotide polymorphisms rs5743899 and, rs3750920 in patients with and, without *P. vivax* malaria

Polymorphism, genotypes and, alleles	*P. vivax* malaria patients	Controls
	n = 301 (%)	n = 255 (%)
TOLLIP rs5743899		
GG	32 (11)	24 (10)
GA	124 (41)	108 (42)
AA	145 (48)	123 (48)
G	188 (31)	156 (31)
A	414 (69)	354 (69)

Polymorphism, genotypes and, alleles	*P. vivax* malaria patients	Controls
	n = 319 (%)	n = 263 (%)
TOLLIP rs3750920		
CC	66 (21)	68 (26)
CT	134 (42)	132 (50)
TT	119 (37)	63 (24)
C	133 (42)	134 (51)
T	186 (58)	129 (49)

Polymorphism, genotypes and, alleles	*P. vivax* malaria patients	Controls
	*p* value	OR [95% CI]
Genotypes and alleles comparisons TOLLIP rs3750920		
CC vs. TT	0.004	1.95 [1.23–3.07]
CC + CT vs. TT	0.0005	0.52 [0.37–0.76]
TT vs. CT	0.002	0.53 [0.36–0.79]
T vs. C	0.002	0.69 [0.55–0.87]

The study examined whether the genotypes of both SNPs were correlated with parasitaemia. Parasitaemia was determined as elsewhere by quantitative Real-Time PCR [25]. Parasites loads were available for only 205 patients with *P. vivax*-infection and ranges from 1 to 158,777 copies/µL. Low parasitaemia was considered to be equal or below to the median (183 copies/µL). None of the genotypes correlated to either low or high parasitaemia as shown in Table 3.

**Table 3:** Analysis of association of the genotype of polymorphism in *TOLLIP* and studied with variables in *Plasmodium vivax* infection

Polymorphism and, genotypes	*P. vivax* malaria patients		OR (IC 95%)	χ^2^	(p) value	
	Low parasite load	High parasite load				
	n = 103 (%)	n = 102 (%)				
TOLLIP 899 (rs3750920)						
G/G	8 (8)	11 (11)	0.738 (0.423–1.287)	1.15	0.283	G/G + G/A vs. A/A
G/A	39 (38)	28 (27)	0.522 (0.186–1.466)	1.55	0.213	G/G vs. G/A
A/A	56 (54)	63 (62)	0.638 (0.349–1.168)	2.13	0.144	G/A vs. A/A
TOLLIP rs3750920						
C/C	35 (34)	37 (36)	0.808 (0.434–1.506)	0.45	0.501	C/C vs. C/T
C/T	48 (47)	41 (40)	0.712 (0.345–1.470)	0.85	0.357	C/T vs. T/T
T/T	20 (19)	24 (24)	1.135 (0.535–2.408)	0.11	0.741	C/C vs. T/T

## Discussion

Despite an increasing amount of research on malaria, the molecular mechanism that influences the clinical outcome of the disease is poorly understood. In endemic areas, only a fraction of *Plasmodium*-infected individuals progress to clinical manifestations while the rest remains asymptomatic. Many may also have recurrent infections or malarial episodes in contrast to their neighbors. Altogether, these suggest that the host genetics may play an important role in the clinical outcome and, the identification of genes involved in either susceptibility or resistance to *Plasmodium*-infection is of utmost interest to the molecular understanding of the disease. Vaccine design may take into account the host genetics.

TLRs upon recognizing invading pathogens orchestrate innate immune responses through the induction of chemokines and inflammatory cytokines to check the parasite. Several polymorphisms present in TLRs genes and, genes involved in its pathways are associated with various infectious diseases [26, 27]. *Plasmodium* GPI is recognized by various cell surface TLRs [15] and, provokes severe malaria symptoms in mice [16]. A perfect control of *Plasmodium* infection by the host requires a well tune immune response sufficient to restrict the multiplication of the parasite and also to avoid an excess activation of the TLRs intracellular signaling that may lead to an exacerbation of pro-inflammatory cytokines such as TNF, IL-6 and, IFN-γ injuring the host tissue.

Two polymorphisms, rs5743899 and rs3750920, in the *TOLLIP* gene that is a negative regulator of TLRs signaling, are associated with tuberculosis in a Vietnamese population [20] and with cutaneous leishmaniasis in the Amazonas state, Brazil [22]. The current study shows that rs3750920 but not rs5743899 is associated with malaria. The rs3750920 T allele is here related to susceptibility to malaria and, is in line with the association with cutaneous leishmaniasis [22] and, with leprosy [23, 28] but in contrast to tuberculosis [20].

TOLLIP diverts the pro-inflammatory cytokine response after TLR-2 and, TLR-4 signaling into an anti-inflammatory response that is characterized by an increased IL-10 and, a decreased IL-6 and TNF expression in peripheral blood monocytes [22]. One study showed that the rs3750920 TT genotype is associated with higher levels of TOLLIP RNA compared to the CC genotype but not with levels of IL-10 or IL-6 [23]. Here the study shows that individuals homozygous for the rs3750920 T allele have twice the chance of developing malaria. It is highly plausible that the diversion of the pro-inflammatory to anti-inflammatory response in carriers of the rs3750920 TT genotype may expose the individual to a higher risk as TNF and, IL-6 cytokines are important early in infection to keep the parasite in check. The genotypes of both SNPs did not correlate to parasitaemia. The rs3750920 may probably do not have an influence on the parasites but mostly on cytokines expressions.

This study has several limitations. Firstly, the sample size of the study participants is small and although the level of the association with malaria is high, it needs validation with a larger sample size. Furthermore, it can be argued that the association to malaria may be spurious as this Amazonian population is a miscegenation of African, European and, Amerindian origin. The controls were properly selected to match the patients’ ethnicity and, are from the same area of endemicity as the patients. Moreover, the frequency of the genotypes of rs5743899 which is very close to rs3750920 was similar in both patients and controls ruling out a spurious association. Secondly, there were no patients with different clinical manifestations, such as severe, mild and asymptomatic malaria, to perform intra-comparison to denote which allele is related to severe malaria. Lastly, plasma cytokines levels were not assayed to correlate with the genotypes of the SNPs.

## Conclusion

The present findings suggest that genes involved in the TLRs-pathway may be involved in the pathogenesis of malaria. TOLLIP interacts with TLR-2 and, TLR-4 to skew the pro-inflammatory to anti-inflammatory response. The association of the *TOLLIP* rs3750920 T allele with susceptibility to malaria further provides evidence that genetic variations in immune response genes may predispose individuals to malaria. Further studies are needed in other vivax regions to confirm whether this allele can be used as a prospective genetic marker for vivax malaria in endemic areas in the future.
